# Inflammation and Abscess of the Uterine Appendages

**Published:** 1849-01

**Authors:** 


*Inflammation and Abscess of the Uterine Appendages.* By Dr. Bennet.—The almost universal view of our profession, that this disease is all but characteristic of the puerperal state, and very rarely occurs under other circumstances, our author considers as very far, indeed, from the truth. The disease is not at all rare in the non-puerperal state, and is often confounded with acute or chronic metritis, iliac abscess, or some other pelvic lesion. In the puerperal form of this affection, and owing probably to the increased qnantity of fibrin contained in the blood, there is a greater tendency to active inflammation. Hence, if the structures contained in the lateral ligaments are attacked with inflammation it has a tendency to spread to the peritoneal folds and the surrounding tissues, giving rise to the formation of large pelvic inflammatory tumours, abdominal adhesions, intestinal perforations, &c., often ending in death. In the non-puerperal inflammations the purulent collections are more limited; the inflammation seldom attacks the peritoneum, and the abscess generally disappears in a latent manner, bursting into the rectum or vagina.

The most frequent seat of this inflammation is the cellular tissue which separates the peritoneal folds and surrounds the ovaries, round ligaments, and Fallopian tubes. It may be produced by any cause which exaggerates the vitality of the uterine system. It may occur in connexion with ulcerative disease of the cervix, or from a severe fall.

The swelling to which it gives rise may often be felt above the pubes, but an accurate diagnosis can be made only by a vaginal examination. The whole may disappear by resolution, but more generally it ends by suppuration, and the discharge of pus by the rectum, vagina, bladder, or through the abdominal parietes.

The treatment of the acute stage is the same as in ordinary phlegmonous inflammation, only it requires to be more active. Bleeding, leeches, cathartics, and mercurials are the chief means to be resorted to. Dr. Bennet places most reliance on the repeated application of leeches to the abdominal parietes, immediately over the seat of inflammation. In the chronic stage, when the parts are not so tender as at first, the application of leeches internally is very useful.—*Lancet*, Feb. 5, 1848.

